# Photodynamic therapy using talaporfin sodium for grade 2/3 meningiomas: a preliminary study of safety, feasibility, and clinical outcomes

**DOI:** 10.1007/s11060-026-05550-2

**Published:** 2026-03-31

**Authors:** Shinjiro Fukami, Jiro Akimoto, Kenta Nagai, Nobuyuki Nakajima, Norio Ichimasu, Ken Matsushima, Sho Onodera, Yuki Saito, Michihiro Kohno

**Affiliations:** 1 Department of Neurosurgery, Tokyo Medical University, 6-7-1 Nishishinjuku, Shinjuku-ku, Tokyo, 160-023 Japan; 2 Department of Neurosurgery, Kohsei Chuo General Hospital, Tokyo, Japan

**Keywords:** Grade 2/3 meningioma, Photodynamic therapy, Talaporfin sodium, Surgical resection, Recurrence

## Abstract

**Purpose:**

Atypical and anaplastic (grade 2/3) meningiomas are associated with high recurrence rates and lack effective systemic therapies other than radiotherapy. Photodynamic therapy (PDT) using talaporfin sodium (TPS) generates selective cytotoxicity extending beyond resection margins and may improve local tumor control. Objective is to evaluate the feasibility, safety, and clinical outcomes of additional intraoperative PDT using TPS for grade 2/3 meningioma patients.

**Methods:**

Nineteen patients with grade 2/3 meningiomas who underwent resection together with PDT at our institute between 2015 and 2025 were retrospectively analyzed. PDT was performed 22 to 26 h after intravenous TPS administration. Tumor location, pathology, Simpson resection grades, history of radiation therapy (RT), and outcomes were reviewed.

**Results:**

Of the 19 patients, 18 had recurrent tumors, and 10 had prior RT. Tumor sites included skull base (8 patients), parasagittal sinus (6), convexity (2), falx (1), subcutaneous (1), and intraventricular (1). Histology revealed grade 2 (12 patients) and grade 3 (7), with Simpson I (3), II (6), III (2), and IV (8) resections. Median progression-free survival was 12.5 months, and median overall survival after PDT was 54.5 months. The prognosis was more favorable in RT- naïve patients than in those with a history of prior RT before PDT.

**Conclusion:**

PDT combined with resection is a safe adjunctive strategy for grade 2/3 meningiomas. Our preliminary data suggest that its efficacy is limited in post-radiation recurrences, but it may delay disease progression when applied before RT. PDT should hence be considered as a therapeutic option for grade 2/3 meningiomas.

## Introduction

Meningiomas are the most common primary intracranial tumors, accounting for approximately one-third of all central nervous system neoplasms. Whereas the majority are benign (World Health Organization (WHO) grade 1), atypical (WHO grade 2), and anaplastic (WHO grade 3), meningiomas comprise approximately 20% of all meningiomas and are associated with aggressive biological behavior, frequent recurrences, and unfavorable clinical outcomes [1, 2]. The standard management strategy for these high-grade tumors includes maximal safe resection, often followed by postoperative radiation therapy (RT) [3]. However, even with gross total resection and adjuvant RT, recurrence rates remain high, with reported median survival times ranging from less than 2 years to more than 5 years, depending on the extent of resection and the use of RT [4–7]. In a previous report, the five-year survival rate for the group consisting of both grade 2 and grade 3 meningiomas was approximately 60% overall [6]. Chemotherapy and molecular targeted therapies, such as temozolomide, bevacizumab, and erlotinib have been investigated, but none have demonstrated consistent efficacy [8]. Recently, a multinational prospective randomized clinical trial of trabectedin for grades 2 and 3 meningiomas with stringent inclusion criteria was performed [9]. However, trabectedin did not improve the progression-free survival (PFS) or overall survival (OS) of these patients, and was associated with higher toxicity than local standard care, such as hydroxyurea or bevacizumab. Therefore, new therapeutic strategies are urgently needed.

Photodynamic therapy (PDT) has emerged as a potential adjunct to surgery in the management of malignant gliomas [10, 11]. PDT involves the systemic administration of a photosensitizer followed by local illumination with a laser of a specific wavelength. Upon excitation, the photosensitizer generates reactive oxygen species (ROS) particularly singlet oxygen, leading to the selective destruction of tumor cells while sparing the surrounding normal tissue [12]. Importantly, the cytotoxic effects of PDT can extend several millimeters beyond the resection margin, potentially targeting microscopic residual disease [13].

In Japan, talaporfin sodium (TPS; Laserphyrin^®^, Meiji Seika Pharma Co., Ltd.), which is a chlorophyll-derived photosensitizer with an absorption peak at 664 nm, has been approved by the national health insurance system for the treatment of not only malignant gliomas but also all primary malignant brain tumors since 2013. Clinical trials have shown encouraging results for glioblastomas, with improvements in local control and acceptable safety profiles [10, 11, 14]. Application of PDT to meningiomas has been less extensively studied, but preclinical study reports suggest potential efficacy. At our institution, we have incorporated PDT into the surgical management of malignant brain tumors, including gliomas and meningiomas, since 2015. In this study, we retrospectively analyzed the outcomes of 19 patients with WHO grade 2/3 meningiomas treated with surgical resection combined with PDT using TPS over a 10-year period, to evaluate its feasibility, safety, and clinical outcomes.

## Materials and methods

### Study design and patient selection

This was a retrospective cohort study conducted at Tokyo Medical University Hospital. Between 2015 and 2025, a total of 180 patients with malignant intracranial tumors, mainly malignant glioma, underwent surgical resection combined with PDT. Among them, 19 patients were histologically diagnosed as having WHO grade 2 or grade 3 meningioma, and were included in this analysis. Inclusion criteria of the patients were as follows: (1) had a tumor that was histologically confirmed as grade 2 or 3 meningioma according to the WHO 2021 classification, (2) the tumor was resectable to some extent, and (3) they had undergone PDT with TPS in conjunction with surgery.

### Surgical procedures and postoperative therapy

All patients received intravenous TPS (40 mg/m²) 22 to 26 h before surgery. After tumor resection, PDT irradiation was delivered to the dural attachment site and, when appropriate, to the adjacent brain parenchyma. Irradiation was performed using a semiconductor laser at 664 nm, with an energy density of 27 J/cm² at an output power density of 150 mW/cm², while avoiding overlaps of the irradiation sites. In cases with narrow surgical corridors or overhanging structures, a custom-designed PDT mirror (PDT mirror^®^, Yufu Itonaga Co., Ltd.) was utilized to reflect and distribute the laser beam effectively [15].

The extent of resection was determined according to the Simpson grading system. Simpson grade I is defined as complete resection including the dural attachment and abnormal bone, grade II as complete resection with coagulation of the dural attachment, grade III as complete resection without coagulation of the dural attachment, and grade IV as incomplete resection. Recurrence was defined as the presence of a new tumor on magnetic resonance imaging (MRI) after grade I/II/III resection, or enlargement/progression/growth of a residual tumor on MRI after grade IV resection.

### Data collection and statistical analysis

Clinical data included patient demographics, prior treatment history (including RT), tumor location, tumor size, extent of tumor resection (Simpson grading), number of sites treated with PDT, pathological grading of meningioma, Ki67 staining, adjuvant treatment, and survival outcome. Follow-up imaging was performed every 3 to 6 months. PFS and OS were calculated from the date of PDT. Survival curves were estimated using the Kaplan-Meier method. Comparisons between subgroups (with vs. without prior RT, pathological grading, extent of resection, Ki67 index, and number of previous resections) were performed using the logrank test. Multivariate analysis was performed using the Cox proportional hazards model. Differences between 2 groups based on RT before PDT, age, duration of illness prior to PDT, tumor size, and differences in survival status were analyzed using the Mann-Whitney test or the logrank test. Statistical analyses were performed using GraphPad Prism 5 software (GraphPad Software, Inc.) and IBM SPSS Statistics version 31 software (IBM Corp.). A statistically significant difference was defined as a *p*-value of less than 0.05.

## Results

The study cohort consisted of 19 patients (5 men and 14 women), with a mean age of 63.4 years. The mean follow-up period was 26.9 months (2–122.2 months). The details and disease courses of all of the patients are shown in Table 1. Eighteen (95%) patients had recurrent tumors, and 10 patients (53%) had undergone prior RT. Of the 18 patients with recurrence, 8 had previously undergone two or more resections. The main lesion of the tumors was as follows: skull base (*n* = 8), parasagittal sinus (*n* = 6), convexity (*n* = 2), falx (*n* = 1), subcutaneous (*n* = 1), and intraventricular (*n* = 1). Pathological analysis revealed WHO grade 2 tumors in 12 patients, and grade 3 tumors in 7 patients. Ki67 labeling index ranged from 5% to 90%. Simpson resection grades were I (*n* = 3), II (*n* = 6), III (*n* = 2), and IV (*n* = 8). Ten patients received postoperative RT following PDT. No PDT-associated complications such as photosensitivity reactions, wound healing delay, or neurological deterioration were observed in the perioperative period. Recurrence occurred in 10 patients, three of which underwent re-resection combined with PDT. The prognosis for all patients was as follows: median PFS (mPFS) after PDT was 12.5 months (95% confidence interval [CI]: 6.7–18.3) and median OS (mOS) was 54.5 months (95% CI: 0.0–114.3). One-year PFS and OS rates were 50.0% and 68.8%, respectively. For the 18 patients with recurrence (excluding the patient of case 7 with a primary tumor), the mPFS and mOS were 12.5 months (95% CI: 6.1–18.9) and 54.5 months (95% CI: 0.0–123.7), respectively. In addition, the mOS from the time of the initial treatment for all patients was 190.2 months (95% CI: 55.4–325.0), which is about 15 years.

**Table 1:** Clinical features of the 19 patients with grade 2/3 meningiomas treated by photodynamic therapy (PDT)

Case	Age (years)	Gender	Duration of illness prior to PDT(months)	Number of tumor resections up to PDT	History of radiotherapy	Tumor location	Tumor size (mm)	Simpson grade	No. of PDT treatments	Pathological meningimoa grading	Ki67 index (%)	Adjuvant treatment	PFS (months)	OS (months)	Recurrence	Patient status at the time writing
1	65–69	F	203.0	1	STI x3	Parasagittal	39	III	x4	2	17	(–)	12.5	54.5	(+), re-PDT	Deceased
2	50–54	M	26.3	1	(–)	Parasagittal	43	II	x3	3	40	CRT	(–)	122.2	(–)	Alive
3	75–78	F	402.0	3	(–)	Middle fossa	34	II	x4	2	10	(–)	23.3	83.3	(+), re-PDT	Deceased
4	55–59	M	181.6	2	STI x4	Cerebellopontine angle-Meckel cave	70	IV	x3	3	70	STI	4.8	9.1	(+)	Unknown
5	50–54	F	183.1	5	STI x2, CRT	Multiple subcutaneous	41	II	x29	3	90	CRT	4.9	7.1	(+)	Deceased
6	65–69	F	45.4	2	IMRT	Cerebellopontine angle and convexity	42	IV	x12	2	20	(–)	15.3	15.3	(–)	Deceased owing to airway obstruction
7	65–69	M	0	0	(–)	Parasagittal-subcutaneous	99	IV	x28	3	70	CRT	8.0	21.6	(+), re-PDT	Deceased
8	45–49	F	26.0	1	CRT	Middle fossa-cerebellopontine angle	38	IV	x10	3	30	STI	11.2	24.1	(+)	Unknown
9	85–89	F	28.7	1	STI	Parasagittal	53	III	x9	2	10	(–)	10.3	10.3	(–)	Deceased owing to breast cancer
10	45–49	F	83.6	1	(–)	Middle fossa-pterygoid fossa	45	IV	x7	2	5	IMRT	(–)	45.2	(–)	Alive
11	40–44	F	53.3	1	(–)	Parasagittal	25	II	x4	3	15	CRT	(–)	32.6	(–)	Alive
12	85–89	F	32.7	1	STI	Middle fossa-temporal-orbit	41	II	x10	2	20	STI	4.5	26.0	(+)	Alive
13	60–64	M	76.2	2	IMRT x2 STI	Multiple, falx and sphenoidal ridge	59	IV	x19	2	20	(–)	2.0	4.6	(+)	Deceased
14	55–59	F	40.7	2	(–)	Convexity	12	I	x15	2	NA	(–)	17.0	17.0	(+)	Alive
15	75–79	F	84.3	1	STI x4	Multiple anterior skull base	62	IV	x17	3	30	(–)	4.8	8.3	(+)	Deceased
16	45–49	F	107.6	2	(–)	Convexity	34	I	x6	2	20	CRT	(–)	11.9	(–)	Alive
17	55–59	F	37.0	1	(–)	Intraventricle	26	I	x6	2	10	IMRT	(–)	12.0	(–)	Alive
18	65–69	F	3.4	1	(–)	Falx	34	II	x5	2	10	(–)	(–)	3.0	(–)	Alive
19	75–79	M	95.4	3	STI	Parasagittal	22	IV	x9	2	15	(–)	(–)	2.0	(–)	Alive

F, female; M, male; PDT, photodynamic therapy; STI, stereotactic irradiation; CRT; conventional radiation therapy; IMRT, intensity-modulated radiation therapy; PFS, progression-free survival; OS, overall survival; re-PDT, repeat photodynamic therapy

### Subgroup analysis: impact of prior RT

**Fig. 1:**
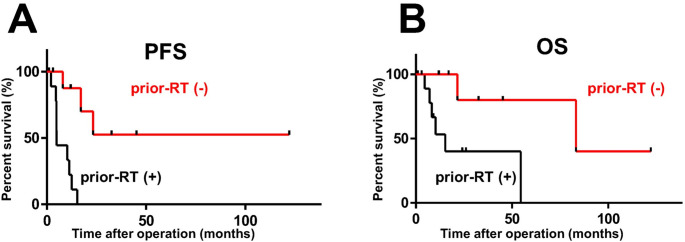
Survival curves of patients with grade 2/3 meningiomas treated by PDT with or without prior radiation therapy (RT). mPFS (**A**) and mOS (**B**) were more favorable in patients who did not undergo prior RT

Patients who underwent RT before PDT were compared with those who did not (Table 2). There were no statistically significant differences between the 2 groups in age, duration of illness, or maximum tumor diameter. On the other hand, the rates of total tumor resection and postoperative RT were higher in the RT-naïve patients than in the preoperative RT group (78% vs. 40% and 67% vs. 40%, respectively). Whereas mPFS remained undefined for RT-naïve patients, the prior RT group showed a mPFS of 4.9 months (95% CI: 4.6–5.2), indicating a statistically significant difference (*p* < 0.001) (Fig. 1A). However, none of the following factors were found to have a significant effect on mPFS: pathological grade (Grade 2 vs. 3 = 15.3 vs. 8.0 months; *p* = 0.785), extent of resection (Simpson grades I–III vs. IV = 17.0 vs. 8.0 months; *p* = 0.183), Ki67 index (≤ 29% vs. ≥ 30%: 15.3 vs. 4.9 months; *p* = 0.229), and the number of previous resections prior to PDT (0–1 vs. ≥ 2 = 11.2 vs. 15.3 months; *p* = 0.420). Multivariate analysis was conducted using a Cox proportional hazards model to identify independent predictors of PFS. The variables included prior RT, extent of resection (Simpson grades I–III vs. IV), and the number of previous resections prior to PDT (0–1 vs. ≥ 2). The results demonstrated that prior RT was a significant and independent unfavorable prognostic factor for PFS, with a hazard ratio (HR) of 16.496 (95% CI: 1.877–144.935; *p* = 0.011). In contrast, neither the extent of resection (HR: 0.972; 95% CI: 0.267–3.542; *p* = 0.96) nor the number of previous resections (HR: 1.466; 95% CI: 0.432–4.978; *p* = 0.540) showed a significant association with PFS. Patients with a history of RT (*n* = 10) had a significantly shorter OS than RT-naïve patients (*n* = 9), with a median OS of 15.3 months (95% CI: 6.4–24.2) versus 83.3 months (95% CI: 0.0–173.0) (*p* = 0.020) (Fig. 1B).

**Table 2:** Comparison of 19 grade 2/3 meningioma patients treated by PDT with or without pre-PDT radiation therapy

	RT (+) (*n* = 10)	RT (−) (*n* = 9)	*p*-value
Operation period (AD)	2015–2025	2015–2025	
Mean age (years)	68.3	57.9	0.11 Mann-Whitney test
Mean period before PDT (months)	95.6	83.8	0.36 Mann-Whitney test
Mean maximum tumor size (mm)	46.7	39.1	0.16 Mann-Whitney test
Tumor pathology			
Grade 2	6	6	
Grade 3	4	3	
Tumor resection			
Simpson grade I, II, or III	4	7	
Simpson grade IV	6	2	
Adjuvant radiotherapy	4 (40%)	6 (67%)	
mPFS (months)	4.9	Undefined	< 0.001 Logrank test*
mOS (months)	15.3	83.3	0.020 Logrank test*

PDT, photodynamic therapy; RT, radiation therapy; AD, Anno Domini, mPFS, median progression-free survival; mOS median overall survival; *, *p* < 0.05

### Two representative cases

#### Case 2: patient with grade 3 meningioma in the parasagittal sinus without prior RT

**Fig. 2:**
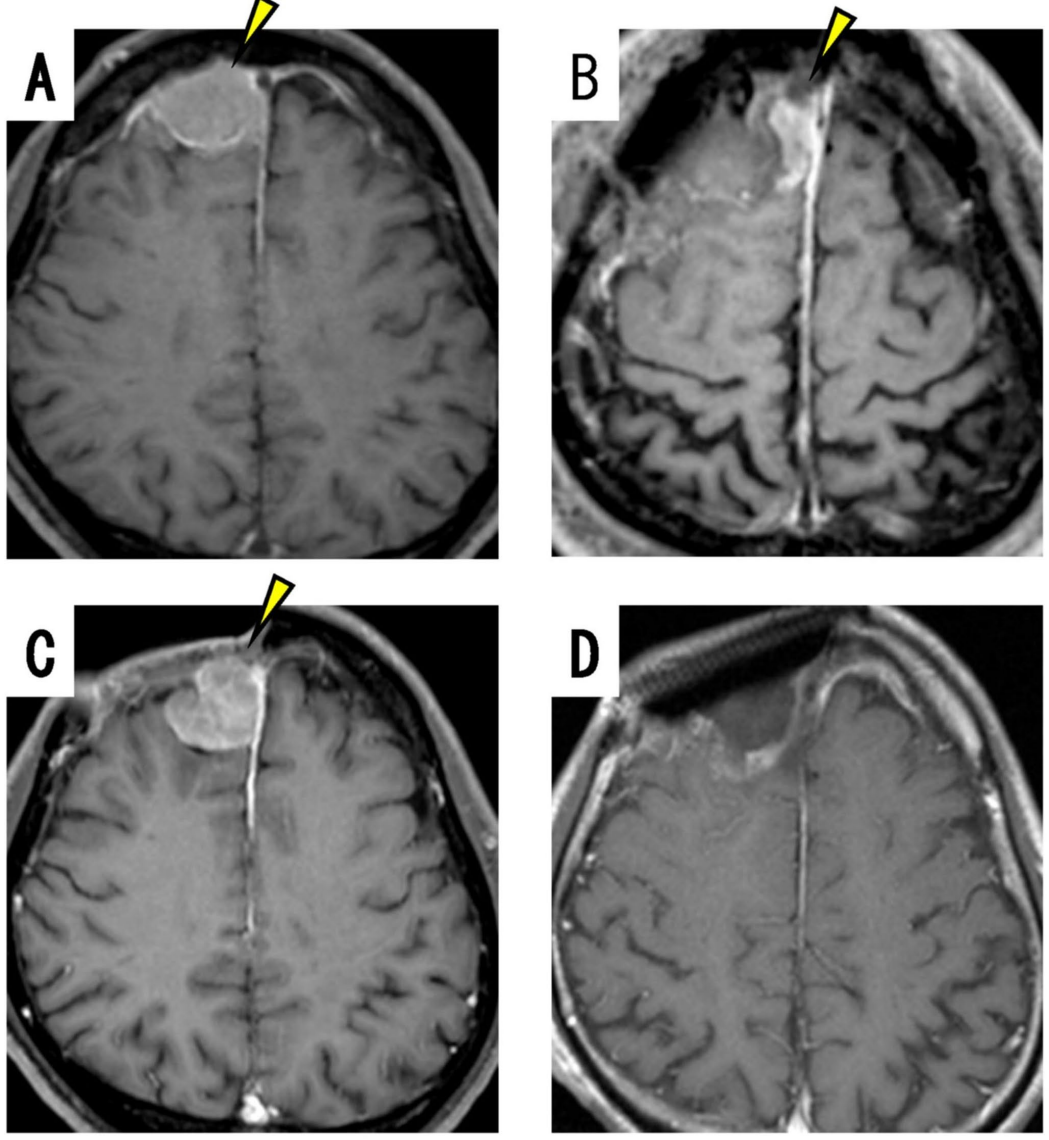
Case 2 (man in his early fifties): grade 3 meningioma in the right parasagittal sinus without prior RT. Gadolinium-enhanced brain MRI taken before (**A**) and 2 days after the initial operation (**B**) displaying the tumor in the right parasagittal sinus (**A**: yellow arrowhead) and the residual tumor (**B**: yellow arrowhead). (**C**) Follow-up MRI taken 2 years after the operation displaying recurrence at the original site (yellow arrowhead). (**D**) Gadolinium-enhanced brain MRI taken 3 weeks after PDT displaying complete removal of the tumor

A man in his early fifties underwent partial resection (Simpson grade IV) for a meningioma located on the parasagittal sinus (Fig. 2A, arrowhead). Pathological examination revealed the tumor to be an atypical meningioma (WHO grade 2). Contrast-enhanced MRI performed 2 days after the initial operation displayed a residual tumor near the sagittal sinus (Fig. 2B, arrowhead). No adjuvant RT was administered; however, the tumor recurred at the initial site 26 months after the initial operation (Fig. 2C, arrowhead). Grade III resection with PDT was performed on the recurrent tumor. Following the resection, PDT was applied to a total of 3 sites within the resection cavity, targeting the dural attachment, including behind the sagittal sinus. The pathological findings from the second resection showed a Ki67 index of 40%, and the tumor was diagnosed as a grade 3 malignant meningioma. MRI performed 3 weeks after the PDT demonstrated complete removal of the tumor (Fig. 2D). One month after PDT, conventional adjuvant RT (60 grays in 30 fractions) was administered. No recurrence was detected on contrast-enhanced MRI performed 122 months after the PDT.

#### Case 12: patient with grade 2 meningioma in the middle fossa-temporal-orbit region with prior RT

**Fig. 3:**
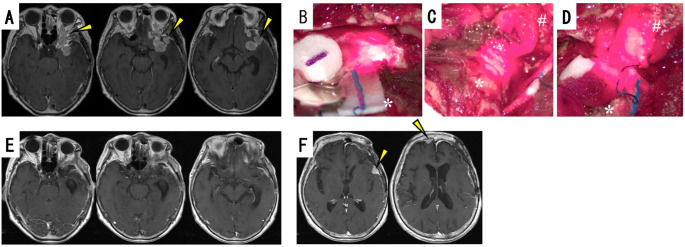
Case 12 (woman in her late eighties): grade 2 meningioma in the left middle fossa-temporal-orbit region with prior-RT. Preoperative gadolinium-enhanced brain MRI (**A**) displaying the tumor extending into the left middle fossa-temporal-orbit region (yellow arrowheads). Intraoperative microscopic view of PDT demonstrating the 664 nm laser-irradiated areas in the dura of the left middle fossa (**B**), the left temporal brain parenchyma (**C**), and the left intra-orbital regions (**D**). Gadolinium-enhanced brain MRI taken 4.5 months after the PDT displaying no recurrence in the areas treated with PDT (**E**), but recurrence was noted in the untreated areas; i.e., the right frontal lobe and the left temporal lobe (**F**, yellow arrowheads). *: left temporal lobe; #: left orbit

A woman in her late eighties presented with memory impairment. Subtotal resection of a left sphenoid ridge meningioma was performed, with the exception of small tumor components surrounding the left middle cerebral artery. Pathological analysis revealed the tumor to be a grade 2 atypical meningioma, and postoperative stereotactic irradiation (STI: 30 grays in 5 fractions) was adjunctively administered. Approximately 2.5 years after the initial resection, recurrence occurred in the middle fossa-temporal-orbit region (Fig. 3A, arrowhead), and vision in the left eye also deteriorated. Grade II resection with PDT was performed on the recurrent tumor. PDT was applied to a total of 10 sites within the dural attachment (Fig. 3B), invaded brain parenchyma (Fig. 3C), and intraorbital region (Fig. 3D). No residual tumor was detected on MRI performed 1 week after PDT. Pathological analysis demonstrated the tumor to be a grade 2 atypical meningioma with a Ki67 index of 20%. Although there was no residual tumor in the targeted area after PDT (Fig. 3E), obvious recurrence was observed 4.5 months after PDT in the right frontal lobe and the left temporal lobe (Fig. 3F, arrowhead), which had not received PDT. Subsequently, STI was performed targeting the recurrence sites, and the patient remains alive 26 months after PDT.

## Discussion

In this study we demonstrated that PDT using TPS combined with surgical resection is safe and feasible for the management of grade 2/3 meningiomas. Since medical insurance coverage for PDT began at our facility in 2014, we have performed PDT on nearly all patients with grade 2/3 meningiomas. Therefore, from 2010 to 2025, there were fewer than 10 patients with grade 2/3 meningiomas at our hospital who underwent resection without PDT. Owing to the insufficient number of patients within our institution to establish an appropriate control group, this study was designed as a preliminary, single-arm retrospective case series. The mPFS from the time of receiving PDT for all 19 patients was 12.5 months, and the mOS was 54.5 months. In addition, the mOS from the time of initial treatment for all patients was 190.2 months (about 15 years). The prognosis for grade 2 or 3 meningiomas varies widely, with OS ranging from less than 2 years to more than 5 years, depending on factors such as the time from diagnosis to resection and whether RT was administered prior to surgery [4–7]. In a recent report on high-grade meningiomas, the extent of resection of the tumor was the primary indicator of a favorable prognosis, both in terms of PFS and OS [16]. Compared with *de novo* (initially grade 2) meningiomas, those that undergo malignant transformation from grade 1 tend to have a less favorable prognosis [17]. In our present study as well, owing to variations in the duration of disease prior to PDT, whether or not preoperative and postoperative RT was performed, and the extent of resection, it remains unclear whether PDT provides any therapeutic benefit. Nevertheless, a mOS of approximately 15 years following the primary treatment appears to be quite favorable. Focusing specifically on the 18 recurrent cases, the mPFS was 12.5 months, and the mOS was 54.5 months. Patients with recurrent grade 2/3 meningiomas show highly variable mOS after salvage treatment, generally ranging from about 2 to 5 years depending on the treatment strategy, including repeat resection, the extent of resection, and the addition of radiotherapy [5, 18–20]. Among our 18 patients with recurrent tumors, 8 (44.4%) received PDT after two or more resections, representing cases of multiple recurrences rather than a first-time recurrence. Specifically, these patients had undergone an average of 2.6 surgical resections (range: 2–5) and 62.5% (5/8) of them had received RT prior to PDT. Given this clinical background, a mOS of 54.5 months is considered favorable. Regarding the prognosis of patients with recurrent grade 2/3 meningiomas following RT, a randomized phase II trial of trabectedin (EORTC-BTG-1320) is frequently cited [9]. That study did not demonstrate trabectedin’s efficacy, the control group in the study appeared to reflect the standard prognosis for recurrent grade 2/3 meningiomas after RT (mPFS: 4.17 months; mOS: 10.61 months) [9]. Regarding the patients in our present study who received RT prior to PDT, their mPFS of 4.9 months and mOS of 15.3 months are numerically higher than those reported in the above trabectedin study. However, direct comparisons should be interpreted with caution due to the heterogeneity of our cohort and the inherent limitations of comparing single-arm data with historical controls from randomized trials. On the other hand, for patients who had not received prior RT, the mOS was relatively favorable at 83.3 months. In this group, the complete resection rate was 78% (7/9), and postoperative radiotherapy was administered in 67% (6/9) of patients. The favorable prognosis might be owing to multiple positive factors, and not solely attributable to PDT. Nevertheless, our results suggest that PDT may provide additional local control for grade 2/3 meningiomas, particularly in RT -naïve patients. Furthermore, our multivariate analysis demonstrated prior RT status as a significant independent predictor of PFS, showing a stronger association than the extent of resection or the number of previous surgeries. This suggests that the impact of prior RT on the efficacy of PDT may differ from that of surgical factors or the chronicity of the disease. However, considering the small sample size and the inherent heterogeneity of this cohort, we recognize that these results should be viewed as hypothesis-generating. On the other hand, it is particularly noteworthy that a reduction in efficacy was observed in patients who had already undergone RT. RT may induce vascular sclerosis and hypoxia, both of which reduce the effectiveness of PDT by impairing oxygen availability [21, 22]. Moreover, failure patterns following PDT occasionally indicated that cerebrospinal fluid dissemination had already occurred. Other considerations include radiation-enhanced intracerebral invasion and biological differences in meningiomas associated with radiation status [23, 24]. On the other hand, basic research has demonstrated that 5-aminolevulinic acid (5-ALA), a drug used in another PDT modality, shows promise in treating meningioma, not only as a photosensitizer but also as a radiosensitizer [25]. TPS also induces physicochemical reactions with X-rays to generate ROS. In pancreatic cancer and glioblastoma models in vitro and in vivo research, the TPS plus X-ray irradiation group demonstrated significant tumor growth suppression compared with the X-ray irradiation-only group, indicating TPS can also function as a radiosensitizer [26]. These findings suggest that PDT should ideally be considered earlier in the treatment sequence, at the first recurrence prior to radiotherapy.

In vitro studies have shown that cultured meningioma cells shrink upon receiving TPS-based PDT, indicating its potential for clinical use [27–29]. The penetration depth of a 664-nm laser into the brain is estimated to be approximately 4 to 5 mm in normal brain tissue, and approximately 10 mm in edematous brain tissue within tumor-infiltrated areas [30]. According to our autopsy data from 3 glioblastoma patients, pathological changes in tissue following PDT occurred within a depth range of 9 to 18 mm from the resection cavity [13]. Therefore, the fact that the cytotoxic effects of PDT extend 5 mm beyond the resection margin of grade 2/3 meningiomas may be particularly advantageous in skull base and parasagittal lesions in which gross total resection is technically difficult. In the present study, PDT was conducted using the same parameters, such as laser power density and energy density, as those established for malignant glioma; however, it remains unclear whether these conditions were sufficient. Given that the in vitro efficacy for meningioma cells was less pronounced than that for malignant glioma cells, exploring higher treatment doses should be considered for future clinical optimization [27]. However, there is a concern that PDT may induce heme oxygenase-1, a heat shock protein, which could potentially trigger treatment resistance; therefore, this approach should be considered with caution [29]. The efficacy of PDT for meningiomas with substantial hyperostosis or bony invasion remains to be established. Currently, it is unclear whether the systemic delivery of TPS achieves therapeutic concentrations within dense bone tissue. Future studies utilizing our newly developed specialized fluorescence microscopy system for photodynamic diagnosis will be essential to evaluate the precise distribution of TPS within the bone, dura, and venous sinus walls, thereby clarifying the therapeutic potential of PDT for complex skull base and parasagittal lesions [31, 32].

The safety of PDT using TPS for malignant gliomas has been well-established in Japan, with a clinical history of more than 10 years. It has been implemented in more than 35 institutions in Japan, and its safety profile is widely recognized, with the exception of minor skin photosensitivity [10, 11, 14, 33]. In this study, PDT for meningioma was performed not only on the brain parenchyma but also on the attached dura and tumor-infiltrated subcutaneous tissue; however, no adverse events, such as skin injury, were observed. Additionally, irradiation was performed on the venous sinus wall where the tumor was attached, but no evident venous sinus thrombosis or venous infarction was observed. Our use of a custom-designed PDT mirror is also of note [15]. This device enables effective illumination in narrow surgical corridors, expanding the applicability of PDT in meningioma surgery. Mirror irradiation was performed for angles of view such as the area behind the sagittal sinus, the skull base, and the ventricle walls, and the convenience of mirror irradiation in meningioma surgeries was acknowledged to be even greater than that for glioma surgeries. It should be noted that the use of a mirror results in power attenuation of the illumination by approximately 30%; therefore, its use should be avoided whenever possible [15].

Limitations of this study include its retrospective design, small sample size, short follow-up period, and lack of a control group. Furthermore, the statistical outcomes, including median survival times (PFS and OS) and HRs in the multivariate model showed reduced reliability due to the broad 95% CIs. Moreover, the heterogeneity of prior treatments and adjuvant therapies complicates interpretation of the results. Nevertheless, the observed survival outcomes and absence of PDT-associated complications support the further investigation of PDT in meningioma treatment. For institutions that are able to safely perform PDT for malignant gliomas, PDT for grade 2/3 meningiomas—for which no effective treatment other than RT is currently available—is considered to be a potential treatment option. Future research should focus on prospective multicenter trials to establish standardized PDT protocols for meningiomas, investigate optimal timing relative to radiotherapy, and explore combination strategies with novel systemic therapies.

## Conclusion

Surgical resection combined with PDT using TPS is a safe and feasible treatment for WHO grade 2/3 meningiomas. Our preliminary findings from this study suggest that PDT might potentially delay tumor progression and may be associated with improved OS, particularly when utilized in RT-naïve patients at the first recurrence. Considering the absence of effective adjuvant therapies other and radiotherapy for grade 2/3 meningiomas, PDT should be considered as an important adjunctive option in their management. Larger prospective studies are warranted to confirm these findings and define the optimal role of PDT in treatment algorithms.

## Data Availability

The data in this study are available from the corresponding author upon reasonable request.
